# Is palliative care a utopia for older patients with organ failure, dementia or frailty? A qualitative study through the prism of emergency department admission

**DOI:** 10.1186/s12913-024-11242-2

**Published:** 2024-07-01

**Authors:** Delphine Bourmorck, Benoit Pétré, Marie de Saint-Hubert, Isabelle De Brauwer

**Affiliations:** 1 Institute of Health and Society, Université Catholique de Louvain, Brussels, Belgium; 2 Department of Public Health Sciences, Faculty of Medecine, University of Liège, Liège, Belgium; 3 CHU-UCL Namur, Yvoir, Belgium; 4 Department of Geriatric Medicine, Cliniques Universitaires Saint-Luc, Brussels, Belgium

**Keywords:** Palliative care, Emergency department, Older patients, Qualitative study

## Abstract

**Background:**

Nearly three out of four older people will use the emergency department (ED) during their last year of life. However, most of them do not benefit from palliative care. Providing palliative care is a real challenge for ED clinicians who are trained in acute, life-saving medicine. Our aim is to understand the ED’s role in providing palliative care for this population.

**Methods:**

We designed a qualitative study based on 1) interviews – conducted with older patients (≥ 75 years) with a palliative profile and their informal caregivers – and 2) focus groups – conducted with ED and primary care nurses and physicians. Palliative profiles were defined by the Supportive and Palliative Indicators tool (SPICT). Qualitative data was collected in French-speaking Belgium between July 2021 and July 2022. We used a constant inductive and comparative analysis.

**Results:**

Five older patients with a palliative profile, four informal caregivers, 55 primary and ED caregivers participated in this study. A priori, the participants did not perceive any role for the ED in palliative care. In fact, there is widespread discomfort with caring for older patients and providing palliative care. This is explained by multiple areas of tensions. Palliative care is an approach fraught with pitfalls, i.e.: knowledge and know-how gaps, their implementation depends on patients’(co)morbidity profile and professional values, experiences and type of practice. In ED, there are constant tensions between emergency and palliative care requirements, i.e.: performance, clockwork and needs for standardised procedures versus relational care, time and diversity of palliative care projects. However, even though the ED’s role in palliative care is not recognised at first sight, we highlighted four roles assumed by ED caregivers: 1) Investigator, 2) Objectifier, 3) Palliative care provider, and 4) Decision-maker on the intensity of care. A common perception among participants was that ED caregivers can assist in the early identification of patients with a palliative profile.

**Conclusions:**

Currently, there is widespread discomfort regarding ED caregivers caring for older patients and providing palliative care. Nonetheless, ED caregivers play four roles in palliative care for older patients. In the future, ED caregivers might also perform the role of early identifier.

**Supplementary Information:**

The online version contains supplementary material available at 10.1186/s12913-024-11242-2.

## Background

The benefits of palliative care (PC) are well established. When given, patients present fewer burdensome symptoms [1, 2], receive less aggressive end-of-life treatments, and are less frequently admitted to emergency departments and hospitals [2–4]. Patients also report a better quality of life, and a better understanding of their disease [5].

However, worldwide, PC is introduced rather late, i.e. only about 19 days before death on average, focuses mainly on patients with cancer and is rarely initiated by first-line workers [6–8]. People suffering from frailty or chronic non-cancerous illnesses are those who benefit least from palliative anticipation [7, 9]. The late introduction of palliative care leads to unnecessary and futile treatments which impact the patient’s quality of life [10, 11].

A number of reasons for the poor and late initiation of PC have been put forward. General practitioners (GP) argue that patients should initiate the Advance care planning (ACP) process [12]. But as patients are unaware of their poor state of health and likelihood of death, they find it difficult to plan their end-of-life care. This is influenced by patients’ trust in their caregiver, their past experiences and their fears or need for control [13]. Moreover, GPs face difficulties to deal with vague patient requests. They also fear depriving the patient of hope, and face the eternal difficulty of finding the "right moment” [12].

Although the responsibility for monitoring older people living with advanced chronic illnesses lies with primary care, the ED is still called upon by primary care workers in a complementary capacity, to manage distressing or acute symptoms which require immediate specialist care. The ED is the technical platform that provides rapid access to advanced diagnostic methods and specific interventions (intravenous administration of powerful painkillers, X-rays, etc.), to medical and paramedical expertise, and that facilitates transfer to other departments [14–16].

Older patients with organ failure, dementia or frailty often suffer from a gradual health deterioration, accompanied by episodes of exacerbated distressing symptoms, which in some cases will lead them to the emergency department (ED) [17]. Even though an ED consultation may be perceived as an undesirable event, it often marks a turning point in the disease, announcing a significative health degradation [18]. As a result, ED carers play a pivotal role in assessing patients’ state of health and referring them to the appropriate care and treatment.

EDs admit up to a quarter of older patients aged of 65 years or over, of which 15% are aged of at least 80 years [19, 20]. Nearly three out of four older patients will use the emergency department (ED) during their last year of life and half of them during their last month of life [21–23]. ED admission can be an opportunity to support the initiation of a palliative care approach for older patients with organ failure, dementia or frailty.

A palliative approach focuses on the patient's quality of life. To this end, it balances examinations and treatments, considering the patient's preferences and wishes. However, ED professionals face a major dilemma in the follow-up of older patients with a palliative profile, if ever identified as such. They have to decide whether carrying on life-prolonging treatments or initiating a palliative approach that includes discussion and decision about intensity of care and some treatment limitation [24]. This is even more difficult for clinicians in the ED who are trained in acute, life-saving medicine. In order to make this type of decision, these clinicians need to be able to identify patients with palliative care needs, which is not an easy task, especially for people with non-cancerous diseases [17, 25, 26]. Nevertheless, a palliative approach is important to ensure appropriate care following patient wishes and goals of care, as well as to avoid poor added value treatments regarding the overall disease trajectory [10, 11].

The literature on palliative care in EDs is emerging and the recommendations are modest and very recent (2017–2021) [16, 27–29]. Despite the limited number of studies and the lack of uniformity in the palliative interventions carried out in the ED, they are showing initial positive effects for the patient and the health care system, such as: improvement of the quality of life without reducing it [30], reduction in length of hospital stays [28], reduction in the number of technical examinations (imaging) and costs [31, 32].

However, the ED ‘s precise role in a palliative care approach, which is not reserved for the very-end-life, is unclear as it seems to vary according to the context, the culture of care and the overall health care system in place.

In this study we aim 1) to gain understanding of the actual role of EDs in palliative care for older patients and 2) to explore potential future roles for them. To achieve these research objectives, we analyse the role of ED professionals in palliative care from their point of view, and from that of primary care professionals, patients and informal caregivers.

## Methods

### Study design

This is a qualitative study including (1) interviews of older patients with a palliative profile and/or their informal carers and (2) focus groups of primary and ED caregivers. We seek to account for the multidimensionality and complexity of the dynamics and logics underlying the studied phenomenon, i.e. the palliative approach in ED for patients aged 75 and over, with an unrecognised palliative profile. We used an inductive and constant comparative approach for data collection and analysis following the Grounded theory process [33, 34].

We collected qualitative data in the French speaking part of Belgium between July 2021 and July 2022. The COREQ criteria supported the report of this qualitative research [35].

### Participants

We considered several types of stakeholders: patients and their informal caregivers on the one hand, primary and ED caregivers on the other. We could not study the ED’s role in palliative care without considering the point of view of primary caregivers. Because of their crucial role in managing older people’s care in community and nursing homes and maintaining continuity of care – particularly after an ED visit and in the overall palliative approach, we included them in the focus groups. We composed a reasoned and diversified sample based on different criteria such as the type of chronic pathology for patients with a palliative profile, gender, profession, and seniority. The description of the study participants can be found in tables [see Tables S1, S2 and S3].

### Data collection

We collected data in two ways: by interviews and by focus groups. The interview and focus group guides used were developed for this study [see Additional files 1 and 2]. We chose to complete face-to-face interviews with patients and informal caregivers due to ethical concerns linked with the sensitivity of the research subject. We chose focus groups for the healthcare professionals’ group. All participants gave informed consent to their participation and authorised the audio recording.

#### Approach for older palliative patients and informal caregivers

We recruited participants purposively, based on a previous study in which older patients were included during an ED consultation [36].

For the current study, patients were recruited during the phone-call follow-up of the aforementioned study. After giving their oral agreement, patients were provided with the document describing the study. We took the time to answer their questions about informed consent and the content of the study. We asked for permission to record them. Patients aged 75 years or over with a palliative profile associated with a non-cancerous disease or frailty were eligible. The palliative profile was defined following the SPICT and explained in Bourmorck et al. (2023). Informal carers were recruited either directly from the previous study during the follow-up, or by a snowball strategy after a patients’ interview.

The field researcher (DB) conducted the interviews mostly at the patients’ private home, respecting their preference. After informal presentations, the opening question was: “Could you tell me about your last visit to the emergency department?”. All the interviews were audio recorded. Field notes were taken after each interview, composed by the main idea developed, the researcher feelings, the process particularities, the researcher questioning and reflexions, key quotes from the participants, unexpected comments, and tracks for the next steps.

#### Approach for primary and ED caregivers

For the focus groups, we recruited nurses and doctors from the primary care settings and emergency departments. For diversity purposes, the recruitment considered local differences in practice and covered the different provinces of French-speaking Belgium, in rural and urban hospitals.

The primary caregivers were recruited by email following a snowball strategy, identified during a patient’s interview or from professional associations. The ED caregivers were recruited within the researchers’ network by emailing the head of ED department of five different hospitals. After a positive response, we used a snowball strategy to recruit participants from the same care network to form the focus groups. This makes possible to highlight the dynamics of a team or network functioning, as well as the organisational and structural work conditions.

Prior to the focus group, exploratory interviews were conducted by field researcher (DB) with each type of professionals, i.e. doctors and nurses from primary care and ED, to specify the focus-group guide and obtain the first descriptive results*.*

Focus groups were conducted in 2 parts: the first part began with “Could you explain how your unit works around older patients with severe chronical conditions?” and was followed by a second question: “What does palliative care mean in your daily work?” in order to get to the heart of research subject more quickly. During the second part of the focus groups, the researchers used clinical vignettes in order to discuss care around specific conditions of older patients with a palliative profile [see Additional file 3]. All the focus groups were audio recorded. Field notes and observations were taken during each focus group by the second researcher (IDB) and discussed directly afterwards, with a threefold aim: 1) to deepen or clarify certain ideas, concepts or statements made by the participants, 2) to fine-tune the interview guide and 3) to carry out a preliminary phase of the analysis process.

Data saturation was discussed with IDB and confirmed with two additional focus group, one from primary care sector and one from ED. Data saturation was reached according to these two criteria: 1) The generic explanation was the same (data saturation related to data collection) [37, 38] and 2) the examples continued to evolve but they no longer added anything to the categories of analysis (inductive thematic saturation focusing on the analysis) [38].

### Analysis

All recorded interviews and focus groups were fully transcribed and were iteratively coded and analysed by the field researcher (DB).

The coding used to anonymise participants in the verbatim transcripts was constructed as follows: “FG” if it was from a focus group (if not, nothing), the working area (“ED” or “Primary”, if not, nothing) followed by the associated number, the type of participant followed by his or her number (“P” for patient, “InformalCaregiver”, “Ph” for physicians, or “N” for nurses). Example: FG_ED1_Ph1 means that the verbatim transcript is from the focus group carried out with the ED team number 1, physician 1.

We used Atlas.ti software to support the first steps of coding. The two first interviews were double coded by an expert in geriatric medicine specialising in qualitative research (IDB). They then met on a weekly basis to support the analysis process and critically review the findings. The whole study process was discussed and followed by an expert in qualitative method.

Analysis was based on the grounded theory process in order to follow an inductive and constant comparative approach. We completed the three first steps i.e. coding, categorising and linking. Firstly, we performed a vertical analysis within each interview or focus group, from which themes and statements emerged, describing and summarising what the participants had to say.

For example, based on the verbatim coming from a primary care focus group, we were able to make those statements:

- Statement 1: 'The organisation of general practice work influences the implementation of palliative care'.
- Statement 2: 'The organisation of general practice work in multidisciplinary collaboration supports the implementation of a palliative approach'.
- Statement 3: 'Conversely, it is more difficult when GPs work alone'.

These statements were summarised under the thematic ‘*General Practice organisation*’.

Secondly, in order to look for communalities in specific experiences, we performed horizontal analysis of the materials from participants with similar profile: a) patients and informal carers, b) ED caregivers and c) primary caregivers. These analyses, allowed the emergence of categories that go beyond description and begin to provide an interpretation of the studied situation, e.g. ‘*Know-do gaps’* [34].

Thirdly, in order to refine and link our initial emerging categories, we performed transversal analysis between the entire material, including field notes and observer notes. This allowed a triangulation that can highlight the convergences and divergences of logics [39]. During the whole process, the main researcher wrote theoretical memo highlighting the links between important themes or categories making it possible to establish relationships between categories. This is how the core category “*widespread discomfort*” emerged, explaining the underlying dynamics about ED palliative care role for older patients.

## Results

### Characteristics of study participants

Five older patients with a palliative profile assessed by SPICT, four informal caregivers, 55 primary and ED caregivers participated in this study. The older patients and informal caregivers covered a diversity of life-limiting conditions, i.e. frailty, dementia and neurological diseases, severe heart, lung, kidney or liver diseases [see Tables S1 and S2].

Most of the nine interviews took place at the patients’ or informal carers’ residence, except one for which the patient preferred to meet at the researcher’s office and one that was performed at the informal carer’s working place. Interviews lasted 40 to 90 min [see Table S1].

Out of the nine focus groups organized, five included primary caregivers and four included ED teams. They took place in the participants’ working place, except one which was conducted by videoconference (Microsoft Teams®). The latter included general practitioners from different provinces available for an online session. The focus groups included between 3 and 15 participants, and lasted 68 to 120 min [see Table S3].

The ED and primary caregivers interviewed by focus groups worked in four of the five French-speaking provinces of Belgium [see tables S3 and S4]. Their years of professional experience ranged from 1 to 48 years. Representatives from both the nursing and physician professions participated, with a majority of general practitioners (*n* = 30). Among primary caregivers, different ways of working were represented (ranging from self-employed and working alone to teams in medical centres). ED caregivers were less available and formed smaller focus groups [see Table S4].

## Main findings

**Fig. 1:**
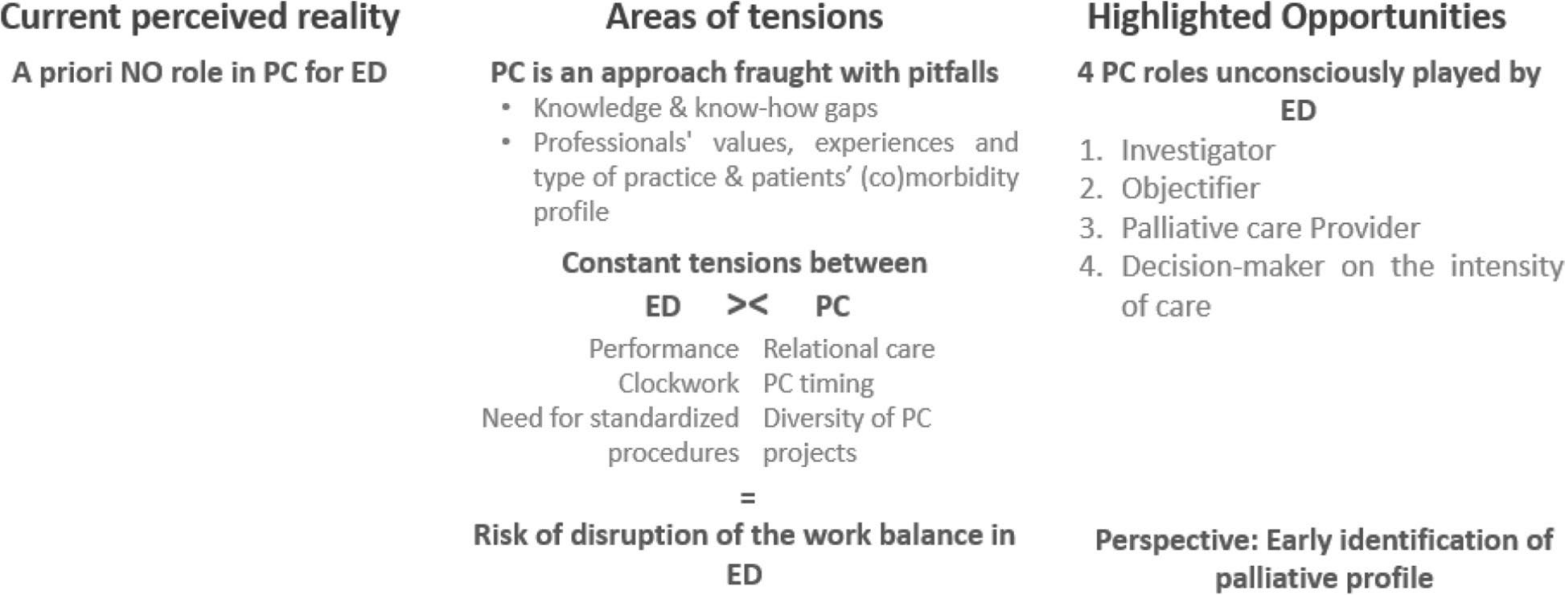
Widespread discomfort in palliative care for older people

A priori, with their current perception of reality, participants did not identify any role in palliative care for the Emergency Departments (ED). In fact, there is *widespread discomfort* (core category) about caring for older patients and providing palliative care (see Fig. 1). This reality is explained by different areas of tensions.

The *palliative care approach is fraught with pitfalls* (category 1) composed by widespread knowledge and know-how gaps (two major themes), where palliative care implementation depends on the type of work organisation, as well as the patients’ type of pathology, and caregivers’ values and experiences (three subthemes) [see the coding tree in Additional file 4].

Moreover, there are *constant tensions between the ED’s routine work and the palliative care approach* for older patients (category 2): 1) performance versus relational care, 2) clockwork versus palliative care requirements, and 3) the need for standardised procedures versus the diversity of palliative care projects (major themes). These tensions lead to a risk of disruption of the work balance in the ED.

Nevertheless, despite this unfavourable context for a palliative approach in the ED with older patients, we highlighted opportunities. ED professionals currently endorse four roles that are part of the palliative approach: the investigator, the objectifier, the palliative care provider and the decision-maker on the intensity of care (major themes). It should be noted that these roles were not all identified by caregivers as part of a palliative approach (palliative care roles unconsciously played by ED, category 3). In addition, participants often mentioned the possibility to contribute to the identification of early palliative profiles as a prospect for the future. These main findings are developed here after.

### Current perceived reality

A priori, in the eyes of most ED and primary caregivers, the ED does not have a role in a palliative approach.

FG_ED4_Ph1: “No, it’s not our place to do it. FG_ED4_N1: It is not our role.”

FG_Primary2_Ph3: “and it is not up to them (in the emergency room) to decide on the final destination.”

Most participants said that it is not the ED caregivers’ role to design an entire Advance Care Plan with an unknown patient in an acute condition. They believe the ED is not the place for global discussions about end-of-live care, it is not the most appropriate and qualified team to achieve a proper advance care planning. Their decisions are limited to the direct ED care. At present times, palliative care in ED takes place at the very end of life. If death is not imminent, it is not their work.

ED_Ph1: “Hmmm (pauses to think). It doesn't seem complex to me. What would concern me, in my opinion, whatever diagnosis we got, is that there was no immediate danger to the patient's life. It's unlikely. So, we will initiate a complex discussion with a patient who says, 'I'm ready. I can die’. In this clinical situation, however, it is not a matter for today … it (end-of-life discussions) will be dismissed by an emergency doctor. He will, if possible, talk about it to the colleague to whom he will refer the patient. Whether it's the GP or, yes, the geriatrician or the pulmonologist. He will pass on the info, but he will not take care of it.”

### Widespread discomfort

In fact, there is *widespread discomfort* (core category) about caring for older patients with a palliative profile, whether in the ED or primary care sector. This discomfort is expressed by the majority of the participating caregivers, across the whole corpus.

FG_ED3_Ph1: “I think the GPs are like us, they are as uncomfortable as we are, and they do as badly as we do. So, they send people when sometimes they shouldn't, because they don't have the time, because they don't always ask themselves the right questions.”

### Areas of tension

#### A care approach fraught with pitfalls

**Fig. 2:**
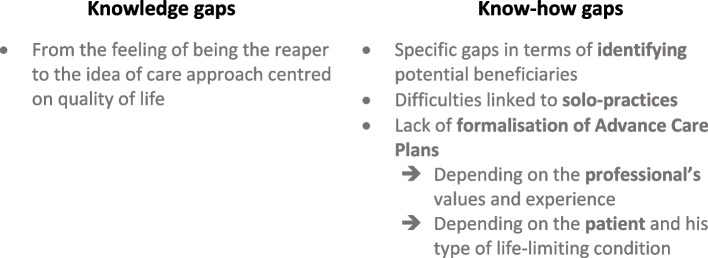
A care approach fraught with pitfalls

The implementation of palliative care for older patients is challenged by *knowledge and know-how gaps* and depends both on the caregiver’s experience and values and on the patients’ profile. These themes are described hereafter (see Fig. 2).

##### Knowledge gaps

The understanding and practice of palliative care evolve at two-tier.

On the one hand, some primary caregivers and most ED caregivers perceive themselves as akin to reapers when initiating palliative care. This responsibility can be burdened with a sense of condemning the patient and a sense of failure. Both caregivers and patients often see palliative care as opposed to the curative approach, and reserved for the very end of life, when there is nothing else to do but to smoothen death. Talking about dying and palliative care remains a taboo. Some patients do not even know what palliative care is and whether they can benefit from it.

P5: “No. I'd rather not hear about it (palliative care). Well, palliative means... ah, I think it's irreversible. The body is tired, the body no longer works normally, whether it's the head, the arms or the feet! The one who's in a cart because he... (he imitates someone bedridden by tilting his head and mouth). As late as possible! That's my only wish [...] in my mind, anyone who goes into palliative care is at the end of his rope. But I've never seen anyone who was treated and cared for in palliative care. I don't suppose we're going to invent that just to plug a hole.”

On the other hand, some primary caregivers describe palliative care as a personalised approach focused on comfort and quality of life.

FG_Primary1_Ph2: “We've gone from end-of-life care when death is imminent to comfort care. But why wait until the end of life to get comfort care? From the moment we know we're entering the process, palliative care should almost begin when we initiate care.”

##### Know-how gaps

Three main know-how gaps emerged from the corpus; the (a) *identification* of potential beneficiaries, the (b) *type of medical practice organisation* and the (c) *formalisation of advance care planning*.

- Identification of potential beneficiaries

The principal know-how gap concerns the *identification* of older patients who may benefit from palliative care. This identification is mostly *intuitive*. The initiation of palliative care *depends on the practitioner*, on their experience, sensitivity and feelings towards the patient’s situation, on their personal values and beliefabout whatis the “right care” for the patient, and on the Belgian law criteria (financial support available if death is estimated within 3 months). Some ED professionals feel fear towards older patients management, while others are not interested in these “cases” or feel useless in “end-of-life situations” and therefore disengage from care. In contrast, others enjoy caring for older patients, whom they see as “interesting medical cases” or “complex challenges”. Some welcome the opportunity to bring back the essence of care: “humanity, dignity, what matters in life”.

FG_ED1 _N1: “It (initiating palliative care) is going to depend on the person. In my case, I might suggest it to the family, and say 'see the attending physician about it or see upstairs'. I might suggest it to my emergency doctor [...] and since there's no well-defined care project, it is set up by such and such a person at such and such a time ... well, it actually depends on who really wants to do it.”

Most practitioners identify patients for PC based on the assessment of comorbidities and severity of chronical diseases. When available, they ask for other opinions (from the general practitioner, the intensive care physician, or the patient’s disease specialist). They refer to death-related probabilities (e.g. number of failing organs). They don’t use tools to support them in the orientation of care and treatment.

The identification also depends on the older *patient’s profile*. Some profiles are less likely to benefit from palliative care, such as patients with *slow health degradation (chronical organ failure), cognitive issues or frailty*. The presence of acute cognitive issues or dementia places ED and primary caregivers in a sensitive position when it comes to deciding on the direction of care. In contrast, caregivers identify patients with a cancer profile more easily as beneficiaries of a palliative care approach.

FG_Primary4_Ph4 : “It's more complicated in the case of neurological diseases, or severe cardiac or respiratory decompensation. Here, the boundaries are much more blurred. [...] When we're still trying this drug for IC (heart failure). Still trying out new drugs for COPD, things like that. Because we think there's still a chance…”

The identification of the palliative profile is also difficult for the informal caregivers and the patients. For some informal caregivers, suffering from slow health degradation and frailty is not linked with palliative needs, because these are not “severe diseases”. Most patients are aware of their illness and their "chronic", “not curable" and sometimes “not operable” quality. However, they do not link those terms with palliative needs. None of the patients who participated in this study identified themselves as having a palliative profile.

P5: “There are illnesses that we know how to cure, there are illnesses that we can cure sometimes, and sometimes not. Cancer, for example. And then there's the chronic illnesses, for which I don't see a solution, and neither do they (caregivers). [...] Nor are they able to say 'I'm going to cure you' .... no, I think the course of this disease is like this (draws a downward sawtooth curve with his finger). Here it's great, but the further along you get, the less capacity you have.”

(b) Type of medical practice organisation

A second know-how gap is related to the organisational practice of care. On the one hand, participants working as *self-employed in a solo *practice reported that implementing palliative care is difficult, even impossible. The need to be constantly available, 24 h a day, is a major constraint for them. Moreover, the time devoted to psychological support and accompanying relatives takes a lot of spacetime in the care process, yet is not valued. Caregivers in ED and in the primary care sector feel that making all the treatment and care decisions alone is too heavy a burden. On the other hand, participants working in a team feel more confident in making those decisions and in implementing PC.

FG_Primary4_Ph4: “If I was a general practitioner all on my own in the depths of my village, without a team around me, with a second line team 40 km away, I might be more reluctant to start the process. But here, I know I can count on the team. That's a support, I'd say, for us as GPs, that multi-level teamwork.”

(c) Poor formalisation of Advance care planning

A third know-how gap is the limited use of Advance care planning (ACP). GPs fear a misuse of ACP, i.e. without re-assessment during a future episode of care, which leads to poor formalisation of a palliative approach. They are also reluctant to share their personal notes. In addition, GPs mentioned difficulties with the translation of patients' wishes into documented medical acts. There is a gap between patients’ ability of looking to the future, including their own wishes, and the translation into medical procedures. Most caregivers ask for the formalisation of the PC project as it is essential for the continuity of care.

P2: “I don't want overtreatment. If there are spare parts, well one can take them, haha ... she said ‘it's written in your file’.”

FG_ED3_Ph1: “Often it's a bit vague, the context isn't clear, patients arrive at the end of their rope (with advanced dementia) with undefined projects and in situations where we must decide whether or not to take action...”

#### Constant tensions between ED routine work and the palliative care approach

Caregivers, patients and informal caregivers expressed constant tensions between ED routine work and the palliative care approach for older patients, i.e. *1) performance versus relational care, 2) clockwork versus palliative care requirements,* and *3) the need for standardised procedures versus the diversity of palliative care projects*. These tensions are described here under.

##### Performance versus relational care

In the ED, caregivers and older patients have different concerns regarding care. Caregivers mostly focus on diagnosis and treatment (see clockwork and standardised procedures verbatim in the next sections). By contrast, patients and their informal caregivers explained that what is key for them is the *care relationship* and benevolence. They come to the ED to release the patient from *burdensome symptoms* linked with their chronic disease (breathlessness, pain, exhaustion, etc.), to manage an *acute illness* (infection, etc.), or to manage a global health degradation related to frailty, sometimes in a context of mental health issue (depression, emotional difficulties, anxiety). They come hoping for a *quick diagnosis*, access to *specific exams or specialist*, an adapted treatment and potentially hospitalisation. But the way they are treated and cared for *is as important as the medical expertise*.

All patients expressed a need for *kindness*, seen as the basis of an optimal care relationship. Kindness means for the patients to feel welcome in the unit (which is one of the first role of ED under Belgian law), to be informed through plain-language communication, and to have their basics needs satisfied. They mentioned the importance to *adapt the rhythm* of the ED with the older patient’s rhythm. These needs for kindness and benevolence are most often expressed in relation to an experience of *ageism* in care.

InformalCaregiver2: “Kindness begins with addressing patients directly. Because in some consultations, they don't ask Mum anymore, they ask me directly. And it's true that this is demeaning for the elderly person ... yes, she may answer less quickly. She'll be less precise. But you can ask the patient first, and then ask the accompanying person afterwards.”

Patients and informal caregivers appreciate a medical approach that *includes patients in decision-making* through accessible communication and explanations.

P2: “And I can ask any question, and get an answer. Whereas before, the doctor would tell you: it's this, it's that, goodbye...”

The *time frame in decision-making* and the presence of an informal caregiver are important to guarantee the patients' autonomy and to respect their preferences for care.

P2: “Yes, and I was the one who decided (not to have shoulder surgery). That was last year, in the spring. He made an appointment for November. He said 'think about it'. I said, 'No, no. When you're 85, I find that... '. And yet, if someone had told me when I fell that they were putting in a prosthesis, they would have put in a prosthesis. If they thought that was the solution... to operate, they would have operated.”

In this study there was no ACP or related documents. All patients explained that they let themselves be taken care of when admitted to the ED. ED physicians were in charge to make all decisions on the treatment and the direction of care.

P3: “In there (in the ED), I let them do whatever.”

##### Clockwork versus palliative care requirements

The ED functions as a *‘Clockwork’*, an industrial-type organisation where every single task is defined in time and space. As explained by ED caregivers, this machinery is designed to fulfil two pivotal roles: 1) maintain, stabilise or restore the patient, and: 2) provide diagnosis and therapeutic guidance. ED physicians endorse a detective role to find all the information needed to solve the patients’ health issue. This works in a context of fluctuating patient flow, which influences patient care. When the flow is high, ED caregivers explained that they focus on the two pivotal roles.

FG_ED1_Ph1: “... Fine-tuned care (implied: linked to a palliative approach and continuity of care) will unfortunately be modulated by the overload we may experience at certain times if I'm overwhelmed, I'll rely on the general information that the nurse gives me, that I have in the file, that I have obtained from the patient. But I won't have the time to phone the family, consult the GP or phone the nursing home. And I won't.”

For most caregivers (ED and primary), discussions about a global palliative care approach are time consuming and are not adequate in the ED due to the acute situation and clockwork. Moreover, multidisciplinary concertation is not easy to organise within the ED. However, some caregivers mentioned that discussions are absolutely necessary and that an ACP can’t replace a discussion in the present moment. The ED routine and exams are not difficult, but to “know how far we go?” in a palliative care approach is more challenging.

FG_ED1_N2: “Our difficulty is that we're on the front line. We have to work with a panel of people around us, and we have to get them to agree. As we were saying, there's the family, there's the specialists, there's the patient … so, it's an uncomfortable position that we are in. In this kind of situation,it’s 'what do we do? where do we start? with whom?' I think that's a real difficulty, because medical management... well, managing examinations, that's easy. It's the same for all patients. But it's more, how far do we go to give the right guidance? Do we hospitalise the patient? Do we keep them at home? Do we…”

When initiated in the ED, the *continuity of palliative care at home* is a challenge for ED clinicians because it is *time consuming*. It is precious to get an outpatient team ready to support the ED. The role of homecare nurses to support ED professional and GP with palliative care organisation is questioned because they are with the patient daily.

For the patients and their informal carers, the continuity of care after an ED admission or hospitalisation is really important. They feel a major rupture when they return home. They mentioned the need for a close follow-up during the first few weeks to ensure the continuity of care.

InformalCaregiver3: “If we could provide people with more precise follow-ups to go by after the patient has left the clinic ... I'd say that from the moment we get to the emergency room, we should determine whether there is a need for follow-up or not [...] I wonder if there is... something that would reassure the families. That we haven't 'just been there' (in the ED).”

##### Need for standardised procedures versus diversity of PC projects

The *diversity of what a palliative care project can be*, the misinformation about the existence of advance care planning (ACP), and the different professional attitudes in palliative care, put ED caregivers in an uncomfortable situation when they have to manage the patient care during ED admission. ED caregivers mention the need for palliative care procedures and alternative of treatments. At the time of the focus group, no palliative procedure existed for ED. Some practitioners mentioned a certain fear and discomfort about dying patient in the ED, especially in relation to the choice of medication and dosage.

FG_ED4_N1: “We're lost when it comes to this whole palliative panel. In the emergency room, we always use the same therapeutic artillery. We're not used to using scopolamine, for example.”

FG_ED1_N2: “We're sitting on the fence. Because in the ED, we're there to perform procedures, and that's sometimes complicated. For the medico-nursing team, saying 'Another doctor has decided it's over', even though it's stipulated, is a complicated thing for some. So what do we do? At what point do we stop the [care]. At what point do you cross the line? Is it applying an antibiotic? Is it suctioning a patient? Is it taking blood? That's... there's really no framework for that. So it really depends on the team you're in.”

These tensions lead to the disruption of the routine work balance in ED.

FG_ED3_Ph1: “Doctors have their own autonomy, but sometimes they tend too much to throw in their assessments and then question themselves, when sometimes it should be the reverse.”

ED_Ph1: “And that's where it gets tricky, it's well "OK, but am I doing what I usually do in front of this patient? ' No! 'All right, I'm not doing what I usually do, but to what extent? ' And to define it? Sometimes, you can't do it because you don't know how to contact people anymore, colleagues ... the patient has a bit of trouble. The patient we have on the ward says, 'Oh well, do what's right. ' Do what's right!? 'but what did your GP say? ' 'to come and see and do what's right.'... Ah ok...”

### Highlighted opportunities

A priori, the participants perceived the ED to have no role in a palliative care approach. Nonetheless, we highlighted opportunities concerning some roles that emergency caregivers perform in the ED and that are part of the palliative approach. They emerged from the focus group during the discussions based on case scenarios (clinical vignettes). The four roles are: *1) the Investigator, 2) the Objectifier, 3) the Decision-maker about the intensity of care, and 4) the Provider of palliative and end-of-life care*. These are roles that they take on but most of these were not initially perceived by ED caregivers as being part of a palliative care approach.

#### The investigator

The ED physician positions himself as an investigator in charge of finding the reasons why the patient arrives with his complaints. He deploys several actions in order to establish his diagnosis and treatment, i.e. anamnesis with the patient and his informal caregiver, auscultation, rapid technical examinations (X-ray, blood test, ECG,…), checking the medical history written in the patient's file and, if necessary, calling the general practitioner or the nursing home.

FG_ED2_Ph1: “If we see the problem, like a fall or delirium or things like that, that's the tip of the iceberg. There's a whole investigation that goes on to find out the underlying cause ... so there's a whole investigation that starts with it.”

This investigator role is hampered by the lack of information available in the patient’s file from the outpatient department, whether it concerns the reasons for admission, the patient's history, the availability of information concerning pre-existing care plans, but also by the difficulties of contacting the professionals who usually care for the patient in an emergency situation.

FG_ED4_N2: “The lack of information leads emergency physicians to take life-sustaining measures, and sometimes there are difficulties when the information arrives, as it is also difficult for team members who suddenly 'pull the plug'.”

#### The objectifier

The objectifier is the one who objectifies. There is a need for a neutral assessment of the patient’s needs. The ED caregivers add value by carrying out a full screening of the patient, from a neutral and external point of view. They assess the patient’s health status and trajectory, with the aim of objectively identifying the causes and the extent of the health problem. They support the general practitioner.

FG_Primary1_Ph1: “We work on the front line with a very high degree of uncertainty. We always treat a little blind. We don't have the X-ray to see if there's a pleural effusion, we don't have the troponin dosage, so we're sailing blindly and sometimes, by dint of sailing blindly, we don't really know where we are anymore. So sometimes hospitalisation (and use of the emergency room) enables us to take stock, to see if what we've done so far is good enough. It's still useful.”

#### The provider of palliative and end-of-life care

A priori, ED caregivers have no role in an “early” palliative care approach (early in opposition to their usual last days/hours of care). Still, they mentioned several situations where they perform legitimate palliative care acts, i.e. releasing burdensome symptoms with treatment adaptation (and sometimes treatment limitation), and accompanying the patient towards death

FG_ED3_Ph1: “But I think there's more to it than that. There's pain, there's weakness, so there's clearly a context of acute suffering, which we can do something about. And then there's the request to stop care or at least, well, yes, rather to limit treatment. Which we can listen to and, based on which, as a result, we can direct our care by saying, well, we're not necessarily going to hospitalise him if we can manage it at the nursing home.”

ED caregivers assessed all the domain that compose palliative profile identification (global health degradation and severity of life limiting conditions). But most of them do not identify them as older patients with palliative profile as defined by the SPICT and Belgian law. Sometimes they perform care and treatment following the philosophy of a palliative care approach without naming it palliative care.

FG_ED1_Ph1: “And so, when I'm confronted with a profile that, medically, has no place in aggressive care, intensive care, hemofiltration, intubation, or resuscitation, quite simply, well, I guide the patient, I hear that he wants to fight, that he would like maximalist care. But he'll have his maximalist care adapted to his profile. And I reassure him because, humanely speaking, you always have to give them a little hope, even if the prognosis is bleak: 'It's going to be complicated, but we'll be able to give you antibiotics, we'll do your maximalist treatment upstairs. But if we have to intubate you, that, that's not going to do you much good, it's going to make you even worse."

#### The decision-maker about the intensity of care

In the ED, the palliative approach takes place under *therapeutic limitations* translated in predefined levels of care. ED caregivers do not perceive it as a proper, complete palliative care approach.

FG_ED4_N3: “The palliative approach begins in the emergency room, because the patient will not be referred to an ICU (intensive care unit) but rather to the geriatric unit, which will begin the process with a life project. And if these people come back, then we'll have a project to follow.”

### Perspective: Identification of early palliative profile

The main possible role for the emergency department in a palliative approach, which was expressed by most caregivers, is to trigger an alarm signal, after identifying the patient.

FG_Primary1_Ph3: “This kind of acute event (recourse to ED) must be the gateway to therapeutic projects. And [we must] ask ourselves, 'if it happens again, what do we do?'”

ED_Ph1: “But we could set up alerts. So that we can alert the colleague to whom we're passing the patient. Saying 'there's no choice, you've got to decide, you've got to discuss it' (with emphasis) ... I'd say be aware, palliative care needs to be put in place, and by the time the patient is discharged, it must have been done.”

FG_ED1_N1: “Maybe it's an opportunity, I'd like to say. It's neither the right place nor the right people, but it's the right time (to initiate a palliative approach).”

## Discussion

Little is known about the role of the ED in palliative care. The findings of this empirical study conducted in the French-speaking part of Belgium give insights to 1) gain understanding of the actual role of EDs in palliative care for older patients and 2) explore potential future roles for them. To our knowledge, this is the first study on ED’s palliative care roles in Belgium.

## Main findings

Our findings highlight key elements that are essential to understanding the *widespread discomfort* about palliative care for older patients with life limiting conditions who are admitted in an ED. A priori, the participants perceived that *EDs have no role to play* in a palliative care approach. This approach of care remains little known (*knowledge gaps*) and poorly applied (*know-how gaps*) by most ED caregivers but also by some primary caregivers. These difficulties of palliative care understanding by ED caregivers were also reported in the UK [40]. From this study, we see that from the patient’s and their GP’s side there is a lack of anticipation of future scenarios, an absence of a clearly established therapeutic project that translate their wishes into documented medical acts. Anticipation is essential, however, to guaranteeing patients' autonomy and respecting their preferences regarding care, particularly when they are admitted to EDs.

The implementation of a palliative approach is also influenced by *individual factors* such as the type of patient's pathology, the experience and values of professionals. Karam et al. (2017) and Leysen et al. (2020) already described the poor use of Advance care planning by Belgian GPs, their difficulties for an early identification of palliative profile -especially for patient with non-cancerous disease, and the need of ED caregivers to have access to palliative care plans, which are confirmed by our study [41, 42].

This study revealed *constant tensions* between the ED’s routine and the palliative care requirements; i.e. 1) performance versus relational care, 2) clockwork versus multidisciplinarity and time requirements, and 3) the needs for standardised procedures versus the diversity of palliative projects. The ED’s primary aim is to ensure rapid assessment and acute clinical care to the detriment of a variable implementation of relational care and palliative care, also described in other studies [27]. The time constraints and management of patients flow, which are part of the clockwork, are one of the four improvement priorities for ED palliative care approach in the UK [40]. We highlighted that these tensions between care logics lead to a *disruption in the work balance in ED*.

However, the provision of palliative care in EDs is necessary. The lack of recognition of ED’s palliative care role, the knowledge and know-how gaps, the lack of skill development and the lack of environmental adaptation can maintain the poor implementation of palliative care in ED [27].

### ED’s roles and perspectives

Despite these contextual, structural and individual elements that slow down the implementation of a palliative care approach in EDs, several roles of ED caregivers were highlighted through the use of clinical vignettes during the focus group discussions: 1) Investigator, 2) Objectifier, 3) Decision-maker about the intensity of care, and 4) Provider of palliative and end-of-life care.

Like in other studies, ED caregivers endorse the investigator role to achieve the management of acute illnesses and distressing palliative symptoms like dyspnea or pain for older patients with a palliative profile, but ED caregivers expressed the need for therapeutic alternatives to their usual medication [27, 43]. Nonetheless, their role in realising an entire advance care planning does not seems to meet the ED caregivers skills, nor the right timing, nor the best environment, as also explained by other studies [27, 44]. The discussions that take place and decisions that are made in EDs are limited to the intensity of care, targeting access to intensive care, intubation and cardiopulmonary resuscitation. The brevity of the therapeutic relationship and the lack of accessible information about pre-established care plans are additional factors that hamper discussions about goals of care [40, 45]. Nonetheless, ED caregivers' external and neutral point of view of, as well as the availability of certain exams and material are requested by general practitioners in order *to take a step back* from the patient's situation. The EDs support primary caregivers by making objective assessments of the patient’s needs (global health assessment), providing palliative care, and being available 24/7. This is in line with another study, in which GPs turned to ED caregivers for advice [41]. In French-speaking Belgium, ED is still often used has first-resort instead of community–based generalist and specialist palliative care [46].

In a context in which older patients’ profiles are unknown, one key potential future role for ED caregivers is as identifiers of patients with a palliative profile. This would support the first step in ACP, which is currently not being taken despite the responsibility on the part of every professional to begin a discussion about the end-of-life care. This ED palliative identification can be a wake-up call for other practitioners to start this discussion.

However, continuity of care, particularly when returning home, is seen as an important element of care for older patients and their informal caregivers. But it is not recognised as part of EDs’ role by ED caregivers, who focus primarily on rapid assessment and acute clinical care. The continuity of care as a part of a palliative approach within EDs is also one of the four areas for improvement in the UK [40].

### Implications for clinical practice and research

Our findings show that there is *widespread discomfort* about caring for older patients*,* and thus highlight the importance of making ED and primary caregivers competent and confident in palliative care for patients aged 75 and above*.* This could be achieved by implementing complex interventions that promote behaviour change through education and interprofessional and organisational collaboration [47]. Below, we outline four components that are essential to achieving this: 1) specific training programmes to improve knowledge and know-how in primary palliative care (education), 2) the recognition of the role of EDs in the palliative care approach (collaboration), 3) the improvement of shared communication about advance care planning (interprofessional and organisational collaboration) and 4) the implementation of a palliative care model in EDs with a focus on continuity of care (with the long-term aim of behavioural change) [40, 43, 48].

1. Training in palliative care needs to be emphasised in initial training curricula, as well as in continuous education courses that are tailored for professionals and their working environment, in order to improve knowledge and skills in palliative care [15, 49]. Continuous education should include senior caregivers to encourage a change of culture through their leadership [27, 50]*.*
2. To achieve recognition of the role of EDs in the palliative care approach, it is necessary to clarify each professional role involved in the palliative approach. Clarifying roles will enable better interprofessional collaboration [41]. A previous study focused on the role of GPs in palliative care, highlighting five of them: care planner (anticipating future scenarios), initiator of decisions in acute situations, provider of end-of-life care, provider of support, and decision maker [51]. Further research is needed to clarify the respective, differentiated, and shared roles of EDs and primary caregivers to ensure better quality and continuity of care for the older palliative patients who are admitted to EDs.
3. Primary caregivers, especially GPs, need to be aware of the impact (a) of relinquishing responsibility for advance care planning, (b) of discussions about palliative care and end-of-life wishes and (c) of the unavailability of this information within the patient's clinical record. This information should be available to ED caregivers [27]. Individual factors such as the fear of ACP misuse could be improved by increasing mutual knowledge and trust [41].
4. At present, models for palliative care in the ED exist, but they are poorly implemented [27]. The only likely perspective for EDs in French-speaking Belgium, under current conditions, is to consciously take on the role of *identifying* palliative profiles in older patients who are admitted. The model for action -after the identification- should be further investigated to avoid misuse of palliative care expertise and inappropriate demands on primary caregivers [52]. Palliative care champions in emergency departments is a promising model of care in this context [14, 15, 27, 49, 52]. Palliative care champions are ED caregivers who have acquired expertise in palliative care that includes the four roles we defined. They can act as expert referents within the department, in addition to their initial role as emergency caregivers [15, 49]. Furthermore, care must be taken to implement a model of care that combines geriatric and palliative care to avoid compartmentalised programmes, given the significant overlap between these two models [53]. Particular attention must be paid to elements that can ensure the continuity of care within the model implemented and adapted to the local context of care. These elements include collaborative processes, communication between caregivers from different sectors, and predefined pathways [43, 48].

Currently, more research is needed to define the care pathway for older palliative patients without cancer and to decide at which stages of the process to integrate the emergency room as a necessary, but not principal, contributor to the palliative approach.

Moreover, our findings also raise questions about the changing role expected of EDs. Initially developed as a response to acute problems, EDs are now the gateway to care for complex problems (chronic illnesses, end of life, etc.). So, is our emergency model still adapted to patient profiles? Does it provide a satisfactory response to society's needs, or does it need to be reorganised as a part of the front-line approach?

### Strengths and limitations

#### Study participants

The participants took part in this study on a voluntary basis. There were no clinician-patient relationships and no direct professional relationships between research team and clinicians. The participants were informed of the general aims of the research with the specification that their opinions and experiences are important and that there were no wrong answers. Particular attention was paid to patients and family carers when talking about palliative care, since they did not receive it and were not recognised as having a palliative profile by professionals. DB monitored patients' knowledge and awareness of their health and care during the first part of the interview, which focused on the use of the emergency department. Once the patient's situation was known, the researcher could move on to the second research topic, which concerned their representation of palliative care.

In total, 55 caregivers and five patients and four informal caregivers participated in this study.

The ED focus groups were smaller due to a lack of available staff and included more department heads than front-line nurses. For these groups, there is a potential selection bias: those who are more aware of the topic are more inclined to take part in the research.

The GP focus group (*n* = 15) turned out to be a precious negative case. Researchers were invited at a GP peer reviewed practice group meeting, but GPs were not previously informed of the specific subject of our research. The recruitment was thus unbiased by “interest for the topic” since the participants discovered it at the beginning of the meeting. They all agreed for participation.

#### Research team and reflexivity

Delphine Bourmorck (DB) is a PhD student in Public Health sciences, trained as a nurse specialised in emergency and intensive care. She is a full time research assistant and previously conducted interviews and observations during focus groups in other research projects (COFI, For-Care) [54, 55]. To ensure the quality of this qualitative study, DB was supported by Isabelle De Brauwer (IDB) and Olivier Schmitz (OS) during the whole research for guidance and advice.

The main risks in qualitative research on palliative care and end-of-life include superficiality, counter-transference and the researcher's eviction mechanism in relation to his own experience [56, 57]. To minimise these potential biases we set up an approach based on the principle of reflexivity [35]. The principal researcher (DB) carried out ongoing self-criticism in view of improving the interviews and focus groups (e.g. by notifying impressions, feelings, uncomfortable events), and reflected on fundamental questions such as “*How am I affected by my research subject*?”.

She used an inductive approach, following grounded theory principles. She took a constructivist posture: we can only see part of the truth; what participants expressed is a fraction of a bigger picture and the situation is constantly moving. Her personal characteristics and research posture may have influenced the research.

We used grounded theory as a method, in which we stopped at the linking phase, due to time and funding constraints. Nevertheless, this level of analysis allowed to answer our research questions. The theoretical sampling was only partial, i.e. we decided what data to collect next (adaptation of the interview or focus group guide) but we did not adapt the inclusion of participants nor the method to collect the data. Further research is needed to achieve a theory about the palliative care role of ED for older patients with life-limiting conditions.

## Conclusion

Palliative care for older patients is causing widespread discomfort among healthcare professionals, patients and their informal caregivers. There are still many barriers to the implementation of palliative care in emergency departments, with tensions exacerbated by the context of acute lifesaving practice. However, even though the ED’s role in palliative care is not recognised at first sight, ED caregivers are effectively playing palliative care roles such as provider of palliative care, decision-maker about the intensity of care and objectifier of the patient’s health situation. In the future, French-speaking Belgian ED caregivers might also perform the role of early identifier and support the formalisation of palliative care by a warning signal. Further research is needed to clarify the palliative care roles of primary and EDs’ caregivers in order to guarantee the quality of care, the continuity of care and the complementarity between emergency expertise and primary care expertise. Research on the implementation of a combined geriatric and palliative care model is also required.

### Supplementary Information

Supplementary Material 1.

Supplementary Material 2.

Supplementary Material 3.

Supplementary Material 4.

Supplementary Material 5.

Supplementary Material 6.

Supplementary Material 7.

Supplementary Material 8.

## Data Availability

Data are available on reasonable request to the corresponding author.

## Abbreviations

ACP: Advance care planning
BMI: Body Mass Index
COPD: Chronic Obstructive Pulmonary Disease
DNR: Do Not Resuscitate
ED: Emergency department
GP: General Practitioner
OP: Older Patient
PC: Palliative Care
SPICT: Supportive and Palliative Indicators tool
