# Supplementary material for: Is palliative care a utopia for older patients with organ failure, dementia or frailty? A qualitative study through the prism of emergency department admission
Source: BMC Health Serv Res. 2024 Jul 1;24:773. doi: 10.1186/s12913-024-11242-2 (PMC11218079; doi:10.1186/s12913-024-11242-2)
Supplement: Supplementary file 1 — Supplementary Material 1. [file 12913_2024_11242_MOESM1_ESM.docx]

**Interview guide with patients or informal caregivers**

**Introduction**

Presentation of the researcher and the research project. Presentation of how an interview works. Requests authorisation to record the discussion. Space for preliminary questions. Read together and obtain informed consent.

**Beginning of the interview**

1. **Building confidence**

| **If the interviewee is the patient** | **Informal caregiver** |
| --- | --- |
| How old are you? | How old are you?  How old is the relative you take care about? |
| What is your marital status? | What is your marital status? And the one of the relative you take care about? |
| What illnesses do you have | What illnesses does your relative have? |
| Can you describe the different people who help you look after yourself ? [What do they do specifically?] | How do you help your relative? What do you do specifically? (For how long?)  Can you describe the other people who help your relative with care? |

1. **The story of the last resort in the emergency department**

- Can you tell me about your (relatives’) last admission to an emergency department (ED)? Follow-up questions:

- When did it happen? Who were you with?

- What made you decide to go to the ED? [What was the main reason?]

- How did the decision to go to the ED come about?

- What did you need (and your relative/person involved in day-to-day care)?

- What happened during your admission to the ED?

- What decisions were made in the ED? How were you involved in the decision-making process?

- What are your wishes (or your relatives’ wishes) in terms of care?

- What was difficult for you when you (or your relative) were transferred to the ED/ or during ED stay?

- If the GP was involved in ED admission:

- How was your GP involved in your admission to the ED?

- How did he support you?

- At what specific moment was he involved [before the transfer to the ED? during the admission? for the return home?]?

- What happened next to the ED admission? (after ED discharge)?

- What was your (relative's) experience of ED discharge?

- How did your (relative's) care change after your ED admission?

- How do you feel about this?

1. **Closing questions**

- In the light of this experience, if you had a magic wand, what would you change (regarding the your /your relatives’ care)?

1. **The palliative care approach**

If palliative care has not been discussed previously, wee opened this new subject of discussion.

Thank you for talking about yourself and your experience, it was really rich. I'm going to move on to another part. For this second part, I would like us to discuss palliative care.

- What do you know about palliative care? What does palliative care mean to you?

- What is your image of palliative care?

- Do you know anyone who is receiving palliative care? Can you explain this to me?

- Have you ever received this type of care? If yes, can you explain this to me?

1. **Summary and final reactions from participant**

Thanks for taking part!
