# Supplementary material for: Is palliative care a utopia for older patients with organ failure, dementia or frailty? A qualitative study through the prism of emergency department admission
Source: BMC Health Serv Res. 2024 Jul 1;24:773. doi: 10.1186/s12913-024-11242-2 (PMC11218079; doi:10.1186/s12913-024-11242-2)
Supplement: Supplementary file 2 — Supplementary Material 2. [file 12913_2024_11242_MOESM2_ESM.docx]

**Focus Group guide with professionals’ caregivers**

**Introduction**

Presentation of the researchers, presentation of how a focus group works. Requests authorisation to record the discussion. Space for preliminary questions. Collects informed consents previously received.

**Beginning of the Focus group.**

1. **Building confidence**

Can everyone introduce themselves in turn? (First name, profession, length of time in the team)

1. **Palliative care in their practice**

As announced, we are interested in certain specific needs of patients admitted to the emergency department (ED), and in particular the need for palliative care.

- What does "palliative care" mean to you in your practice?

[Follow-up questions: How does it differ from comfort care/ end-of-life care/and discussions about the end of life? What do you think of Advance Care Planning?]

- What roles do you have with palliative patients (doctors and nurses’ roles)?

- What procedures or guidelines do you follow in your department? Can you give me details or provide me your protocol?

1. **Clinical vignettes**

Now, I would like you to interact with vignettes of older patients presenting to ED. These vignettes are inspired by real-life situations. I will leave you each some time to read this first vignette.

Don't hesitate if you have any questions about the vignettes, even during the discussion.

- What do you think about this patient's use of the ED [how is it appropriate? justified? inappropriate?]

- What do you find more difficult or uncomfortable for this patient to manage in the ED?

- In your opinion, what are the most important things to consider during this patient's admission?

- Do you think there was an alternative to admission to the ED? [if yes, which one?]

- In your opinion, what factors would make you think that this patient could benefit from a palliative care approach? [Would you introduce a palliative care approach for this patient? What are the reasons?]

Whether the participants think that the palliative approach could be initiated:

- What would support you in initiating a palliative care approach for this patient?

- What do you think is feasible in ED about palliative care for this patient?

Follow-up questions if they address the issues:

- What difficulties do you have in initiating a palliative care approach?

- In your opinion, when is the best time to discuss care preferences? For example, when do you think it is appropriate to discuss care preferences, such as limiting treatment, end-of-life issues or setting up palliative comfort care?

1. **Closing questions**

- At present, what role do you think EDs could play in implementing a palliative care approach?

OR [depending on the previous discussion]

- Do you think that EDs have a role to play in setting up palliative care? If so, what role? And if not, why not?

OR

- How can EDs caregivers support primary caregivers in initiating a palliative care approach?

- If you had a magic wand, what would you put in place to initiate a palliative care approach for older patients in the ED?

1. **Moderator's summary and final reactions from participants**

Thanks for taking part!

1. **Moderator-observer debriefing**
