# Supplementary material for: Is palliative care a utopia for older patients with organ failure, dementia or frailty? A qualitative study through the prism of emergency department admission
Source: BMC Health Serv Res. 2024 Jul 1;24:773. doi: 10.1186/s12913-024-11242-2 (PMC11218079; doi:10.1186/s12913-024-11242-2)
Supplement: Supplementary file 3 — Supplementary Material 3. [file 12913_2024_11242_MOESM3_ESM.docx]

**Clinical vignettes used during focus groups**

**Mrs Rose**

Mrs Rose is 89 years old. She lives at home with her son. She has been admitted to the ED due to a fall. She was found at the bottom of her bed this morning. According to her son, she falls regularly. She is less and less independent. Her son has to keep encouraging her to get up and eat. She can no longer dress or wash herself without help.

She has stopped taking her thyroid medication and has severe hypothyroidism, which may explain her current fatigue symptoms. She probably has chronic cognitive problems, but this is unclear and has not been investigated.

She says, "I want to die". According to her son, she has been expressing this wish for several months.

**Mr Lancelot**

Mr Lancelot is 81 years old. He lives in a nursing home and has two children. He presented to the ED this morning with severe respiratory difficulties. The diagnosis in the emergency room was acute pulmonary oedema. This is the third in four months. The patient has suffered from heart failure for eight years. He suffered a myocardial infarction in 2015 and is hospitalised almost every year for treatment adjustments. No intervention is possible for the patient. In the emergency room, the patient complains and says he's fed up with going back and forth from hospital.

**Mrs Wu**

Mrs Wu arrived at the emergency department by ambulance. Her general condition had deteriorated. Her husband explained the situation.

Mrs Wu is 89 years old and lives at home with her husband. A nurse visits to help her with her daily grooming.

She has been suffering from dementia for two to three years, which is having an increasingly marked impact on her daily life. She also suffers from stage four chronic renal failure (renal glomerular filtration <30ml/min).

The diagnosis in the emergency room was acute global cardiac decompensation in the context of inoperable ischaemic valvular and rhythmic heart disease.

**Mrs Danse**

Mrs Danse is 77 years old. Just over a year ago, she was diagnosed with the neurodegenerative disease Charcot (amyotrophic lateral sclerosis). Today, she has come to the ED with her daughter because her condition has deteriorated dramatically. She can barely move, is in severe pain and has great difficulty speaking.

She is being monitored by a mobile palliative care team.

**Mr Tetaz**

Mr Tetaz arrived in the emergency room alone by ambulance. He is 85 years old, a widower, and he lives in a nursing home.

He came to the emergency room with dyspnoea and COPD. The diagnosis was bilateral respiratory syncytial virus pneumonia.

The patient is oxygen-dependent. He suffers from heart failure and is diabetic.

He has had a stroke in the past. He has severe walking difficulties and has fallen several times in the last few months. He uses a rollator to walk short distances from his wheelchair to the bathroom. He is urine incontinent. He is out of breath, his pain is rated at 6/10 and he has significant muscle weakness. He feels very tired.

He says, "I'm ready, I can die".

**Sister Marie-Clarence**

Sister Marie-Clarence arrived in the ED during the night. She presented with an altered general condition and post-dialysis fever. The emergency room diagnosis was staphylococcal sepsis of undetermined origin.

She came from a religious community. She is 79 years old.

She suffers from heart failure, COPD and chronic renal failure, and is on dialysis three times a week. Sister Marie-Clarence can hardly walk any more. She can get up from her wheelchair with help to go to the toilet. On dialysis days, she feels extremely tired. She has a loss of appetite and her BMI is 33.
