# Supplementary figures and images for: Is palliative care a utopia for older patients with organ failure, dementia or frailty? A qualitative study through the prism of emergency department admission
Source: BMC Health Serv Res. 2024 Jul 1;24:773. doi: 10.1186/s12913-024-11242-2 (PMC11218079; doi:10.1186/s12913-024-11242-2)

**Coding tree**


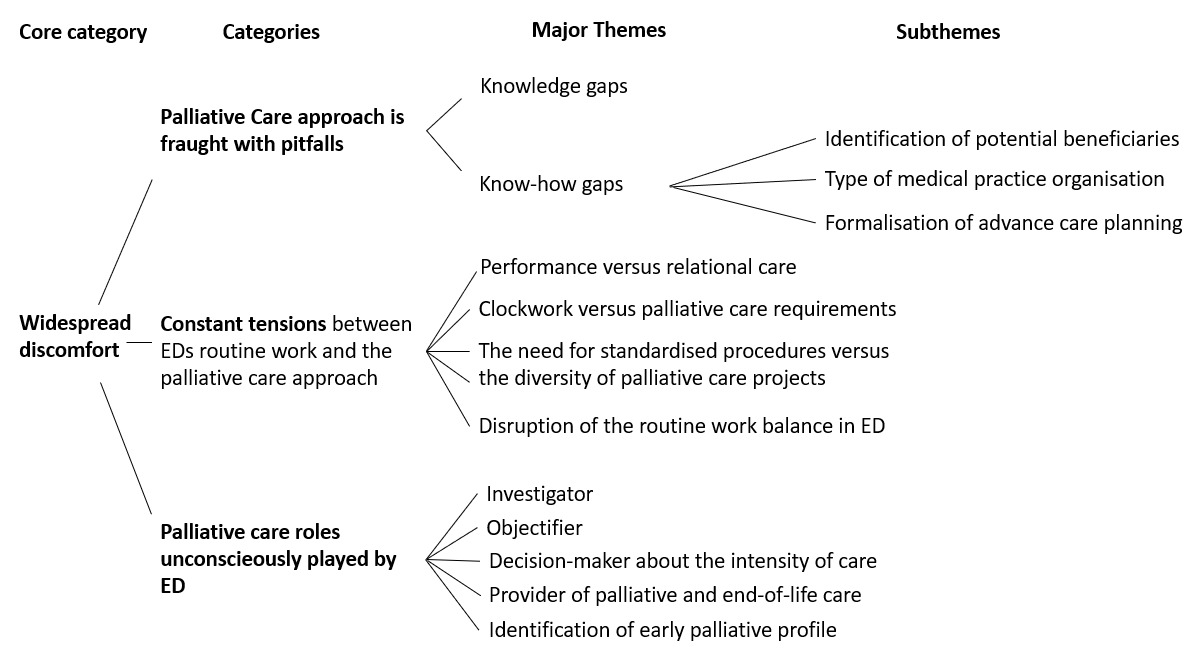

Supplement: Supplementary file 4 — Supplementary Material 4. [file 12913_2024_11242_MOESM4_ESM.docx]
