# Supplementary material for: Is palliative care a utopia for older patients with organ failure, dementia or frailty? A qualitative study through the prism of emergency department admission
Source: BMC Health Serv Res. 2024 Jul 1;24:773. doi: 10.1186/s12913-024-11242-2 (PMC11218079; doi:10.1186/s12913-024-11242-2)
Supplement: Supplementary file 5 — Supplementary Material 5. [file 12913_2024_11242_MOESM5_ESM.docx]

**Table S1 : Description of interviews with older patients and informal caregivers**

| **Interviews**  **n = 9** | **Older patients with palliative profile**  **n = 5** | **Informal caregivers**  **n = 4** |
| --- | --- | --- |
| **Gender**  Male  Female | 3  2 | 1  3 |
| **Age** (years, mean) | 81.6 (min 79 - max 85) | 57.2 (min 47 - max 80) |
| **Living place of the OP cared -** Home | 5 | 4 |
| **Marital status of OP cared**  Married – in partnership  Widow  Single | 3  1  1 | 2  2  0 |
| **Educational level**  Primary school  Secondary school  Tertiary and over  Unknown | 0  3  2  0 | 0  0  3  1 |
| **Principal chronical disease of the OP cared**  Polyarthritis  Severe heart failure  Polypathology : severe kidney disease without dialysis, heart failure, lung failure, cirrhosis, diabetes)  Chronic renal failure on dialysis  COPD stage 4  Severe dementia  Frailty | 1  1  1  1  1  0  0 | 1  0  0  0  1  1  1 |
| **Documentation about palliative care, ACP or DNR document** | 0 | 0 |
| **Linked with the OP cared**  Child  Wife  Friend |  | 2  1  1 |
| **Average length of interview** (minutes) | 53 (min 48- max 58) | 60 (min 40 -max 96) |

Encircled are the associated patients and informal carers, interviews were realised separately.

Palliative profile assessed by SPICT is described in table S2.
