# Supplementary material for: Is palliative care a utopia for older patients with organ failure, dementia or frailty? A qualitative study through the prism of emergency department admission
Source: BMC Health Serv Res. 2024 Jul 1;24:773. doi: 10.1186/s12913-024-11242-2 (PMC11218079; doi:10.1186/s12913-024-11242-2)
Supplement: Supplementary file 6 — Supplementary Material 6. [file 12913_2024_11242_MOESM6_ESM.docx]

**Table S2: Description of the palliative profiles of the older patients according to SPICT assessment**

|  | **General indicators of deteriorating health^1^** | | | | | **Life-limiting conditions^2^** | | | | | |
| --- | --- | --- | --- | --- | --- | --- | --- | --- | --- | --- | --- |
|  | **(1)**  **Unplanned hospital admission** | **(2)**  **Poor performance** | **(3)**  **Depends on others because of increasing health problems** | **(4)**  **Weight loss, low muscle mass** | **(5)**  **Persistent symptoms despite optimal treatment** | **Frailty or dementia** | **Neurological disease** | **Cardiovascular disease** | **Respiratory disease** | **Kidney disease** | **Liver disease** |
| **Patient 1** | **X** | **X** |  |  | **X** | **X** |  | **X** |  |  |  |
| **Patient 2**  **and cared by informal caregiver 2** | **X** | **X** | **X** |  |  | **X** |  |  |  |  |  |
| **Patient 3** | **X** | **X** |  |  |  | **X** |  |  |  | **X** |  |
| **Patient 4** |  |  | **X** |  | **X** |  |  |  | **X** | **X** | **X** |
| **Patient 5**  **and cared by informal caregiver 4** | **X** |  |  |  | **X** |  |  |  | **X** |  |  |
| **Patient cared by informal caregiver 1** |  | **X** | **X** |  | **X** | **X** | **X** |  |  |  |  |
| **Patient cared by informal caregiver 3** |  | **X** | **X** | **X** | **X** | **X** |  |  |  |  |  |

^1^: No patients asked for palliative care or expressed wishes to focus on quality of life (item 6 in SPICT)

^2^: No patients were selected based on a principal diagnose of cancer
