# Supplementary material for: Is palliative care a utopia for older patients with organ failure, dementia or frailty? A qualitative study through the prism of emergency department admission
Source: BMC Health Serv Res. 2024 Jul 1;24:773. doi: 10.1186/s12913-024-11242-2 (PMC11218079; doi:10.1186/s12913-024-11242-2)
Supplement: Supplementary file 7 — Supplementary Material 7. [file 12913_2024_11242_MOESM7_ESM.docx]

**Table S3 : Description of the focus groups with primary caregivers**

|  | **Focus group 1**  n=7 | **Focus group 2**  n=15 | **Focus group 3**  n=3 | **Focus group 4**  n=6 | **Focus group 5**  n=8 | **Total**  n=39 |
| --- | --- | --- | --- | --- | --- | --- |
| **Gender**  Male  Female | 3 4 | 10 5 | 3 0 | 1 5 | 3 5 | 20 19 |
| **Professions**  Physicians  Nurses | 3 4 | 15 0 | 3 0 | 4 2 | 5 3 | 30 9 |
| **Professional seniority** (mean years; min-max) | 24.6 (3 - 40) | 28.2 (5 - 48) | 6 (1 - 12) | 11.7 (3 - 35) | 14.6 (1 - 40) | 20.8  (1 - 48) |
| **Length** (minutes) | 113 | 101 | 96 | 68 | 120 | 99.6 (68 - 120) |
| **Particularities** | Independent fee-for-service doctors  Home care nurses, Nursing-home care nurses and nurses from mobile palliative care teams (second line) | 7 Independent fee-for-service doctors,  7 Independent working in pairs,  1 medical center | 3 Independent fee-for-service doctors working in 3 different provinces  (realised by Teams) | Team from a single medical center | Physicians coming from 2 distinct medical center  Independent home care nurses  Nurses from mobile palliative care teams (second line) |  |
