# Supplementary material for: Is palliative care a utopia for older patients with organ failure, dementia or frailty? A qualitative study through the prism of emergency department admission
Source: BMC Health Serv Res. 2024 Jul 1;24:773. doi: 10.1186/s12913-024-11242-2 (PMC11218079; doi:10.1186/s12913-024-11242-2)
Supplement: Supplementary file 8 — Supplementary Material 8. [file 12913_2024_11242_MOESM8_ESM.docx]

**Table S4 : Description of the focus groups with ED caregivers**

|  | **Focus group 1**  n=5 | **Focus group 2**  n=3 | **Focus group 3**  n=4 | **Focus group 4**  n=4 | **Total**  n = 16 |
| --- | --- | --- | --- | --- | --- |
| **Gender**  Male  Female | 1 4 | 0 3 | 2 2 | 1 3 | 4 12 |
| **Professions**  Physicians  Nurses | 2 3 | 1 2 | 4 0 | 1 3 | 8 8 |
| **Professional seniority** (mean years, min-max) | 12.2 (4 -20) | 15.6 (4 – 31) | 6 (1 – 12) | 30 (30 - 30) | 16.2 (1 - 31) |
| **Length** (minutes) | 118 | 86 | 72 | 110 | 96.5 (min 72 – max 118) |
